# Physiological Condition-Dependent Changes in Ciliary GPCR Localization in the Brain

**DOI:** 10.1523/ENEURO.0360-22.2023

**Published:** 2023-03-13

**Authors:** Kathryn M. Brewer, Staci E. Engle, Ruchi Bansal, Katlyn K. Brewer, Kalene R. Jasso, Jeremy C. McIntyre, Christian Vaisse, Jeremy F. Reiter, Nicolas F. Berbari

**Affiliations:** 1 Department of Biology, Indiana University-Purdue University Indianapolis, Indianapolis, Indiana 46202; 2 Department of Neuroscience and Center for Smell and Taste, University of Florida, Gainesville, Florida 32603; 3 Diabetes Center and Department of Medicine, University of California San Francisco, San Francisco, California 94143; 4 Department of Biochemistry and Biophysics, Cardiovascular Research Institute, University of California, San Francisco, San Francisco, California 94158; 5 Stark Neurosciences Research Institute, Indiana University, Indianapolis, Indiana 46202; 6 Center for Diabetes and Metabolic Diseases, Indiana University School of Medicine, Indianapolis, Indiana 46202

**Keywords:** accumbens, feeding behavior, G-protein-coupled receptors, hypothalamus, obesity, primary cilia

## Abstract

Primary cilia are cellular appendages critical for diverse types of Signaling. They are found on most cell types, including cells throughout the CNS. Cilia preferentially localize certain G-protein-coupled receptors (GPCRs) and are critical for mediating the signaling of these receptors. Several of these neuronal GPCRs have recognized roles in feeding behavior and energy homeostasis. Cell and model systems, such as *Caenorhabditis elegans* and *Chlamydomonas*, have implicated both dynamic GPCR cilia localization and cilia length and shape changes as key for signaling. It is unclear whether mammalian ciliary GPCRs use similar mechanisms *in vivo* and under what conditions these processes may occur. Here, we assess two neuronal cilia GPCRs, melanin-concentrating hormone receptor 1 (MCHR1) and neuropeptide-Y receptor 2 (NPY2R), as mammalian model ciliary receptors in the mouse brain. We test the hypothesis that dynamic localization to cilia occurs under physiological conditions associated with these GPCR functions. Both receptors are involved in feeding behaviors, and MCHR1 is also associated with sleep and reward. Cilia were analyzed with a computer-assisted approach allowing for unbiased and high-throughput analysis. We measured cilia frequency, length, and receptor occupancy. We observed changes in ciliary length, receptor occupancy, and cilia frequency under different conditions for one receptor but not another and in specific brain regions. These data suggest that dynamic cilia localization of GPCRs depends on properties of individual receptors and cells where they are expressed. A better understanding of subcellular localization dynamics of ciliary GPCRs could reveal unknown molecular mechanisms regulating behaviors like feeding.

## Significance Statement

Often, primary cilia localize specific G-protein-coupled receptors (GPCRs) for subcellular signaling. Cell lines and model systems indicate that cilia deploy dynamic GPCR localization and change their shape or length to modulate signaling. We used mice to assess neuronal cilia GPCRs under physiological conditions associated with the known functions of receptors and ciliopathy clinical features like obesity. We show that particular cilia with specific GPCRs appear to dynamically alter their length, while others appear relatively stable under these conditions. These results implicate multiple themes across cilia GPCR-mediated signaling and indicate that not all cilia modulate GPCR signaling using the same mechanisms. These data will be important for potential pharmacological approaches to target cilia GPCR-mediated signaling.

## Introduction

Cilia are nearly ubiquitous, small microtubule-based cellular appendages critical for proper development and homeostasis where they coordinate specific signaling pathways (Reiter and Leroux, 2017). Thus, cilia structure or function defects can result in many disorders with a broad array of clinical features (Reiter and Leroux, 2017). Collectively known as ciliopathies, these disorders are often associated with neural developmental or behavioral deficits. In addition, certain ciliopathies are associated with increased feeding behavior and obesity (Vaisse et al., 2017; Engle et al., 2021; Lee et al., 2022). Altered hypothalamic cilia signaling has been implicated in ciliopathies associated with obesity (Davenport et al., 2007; Loktev and Jackson, 2013; Sun et al., 2021; Wang et al., 2021b,c).

Despite their clinical relevance and an understanding of cilia-mediated signaling in development, little is known about the roles of cilia on terminally differentiated neurons *in vivo* and how they influence mammalian behaviors. A diverse set of G-protein-coupled receptors (GPCRs) appear to preferentially localize to cilia, including specific GPCRs with known roles in feeding behavior and energy homeostasis, such as melanin-concentrating hormone receptor 1 (MCHR1) and neuropeptide-Y receptor 2 (NPY2R) (Berbari et al., 2008a,b; Loktev and Jackson, 2013).

During embryonic development, dynamic localization of signaling machinery and a GPCR (GPR161) to the ciliary compartment in a ligand-dependent manner is critical for proper hedgehog signaling (Mukhopadhyay et al., 2013; Hwang and Mukhopadhyay, 2015; Pal et al., 2016). In addition, *Chlamydomonas* and *Caenorhabditis elegans* use cilia length, shape, vesicular shedding, and receptor localization changes to mediate signaling (Mukhopadhyay et al., 2008; Olivier-Mason et al., 2013; Wang et al., 2020, 2021a). Mammalian cell line data also clearly demonstrate the dynamic localization of ciliary GPCRs as a potential mechanism to mediate signaling, and ciliopathy mutations are associated with deficits in these processes (Ye et al., 2013; Nager et al., 2017; Phua et al., 2017; Shinde et al., 2020).

In mammalian adult homeostasis, less is understood about how cilia mediate GPCR signaling in the CNS. The most well studied examples are the photoreceptor and olfactory sensory neuron cilia, which mediate opsin/rhodopsin and odorant receptor signaling for vision and olfaction (Singla and Reiter, 2006; Berbari et al., 2009). Here, we sought to determine whether cilia GPCR localization, frequency, and length dynamics change within brain regions associated with both the specific GPCR function and ciliopathy-associated clinical features such as obesity. We focused on two ciliary GPCRs: MCHR1 and NPY2R. Both are expressed in the brain, including hypothalamic feeding centers. MCHR1 has also been implicated in sleep and reward (Pissios et al., 2008; Presse et al., 2014; Blanco-Centurion et al., 2019; Dilsiz et al., 2020). To determine whether these GPCRs dynamically localize to cilia *in vivo*, we assessed their localization under different feeding conditions. We hypothesized that cilia GPCRs throughout the CNS would dynamically localize to the compartment based on changes in signaling, similar to other model systems and cell line data.

## Materials and Methods

### Mice

All procedures were approved by the Institutional Animal Care and Use Committee at Indiana University-Purdue University Indianapolis. Adult C57BL6/J mice were obtained from The Jackson Laboratory (stock #022409). Unless identified within the figure (see Fig. 2), all experiments were conducted in male animals. Unless stated otherwise, mice were housed on a standard 12 h light/dark cycle with *ad libitum* food and water.

### Feeding conditions

Fed mice were allowed *ad libitum* access to food, fasted mice had no food overnight (∼16 h), and Refed mice were given 4 h of *ad libitum* access to food immediately after an overnight fast.

### Diet-induced obesity

Mice were fed either a standard chow diet consisting of 13% fat, 58% carbohydrate, and 28.5% protein caloric content (catalog #5001, LabDiet) or a calorie-rich, high-fat diet (HFD) consisting of 60% fat, 20% carbohydrate, and 20% protein caloric content beginning at 8 weeks of age (catalog #D12492, ResearchDiets). Mice were weighed weekly before proceeding to tissue analysis after 11 weeks on these diets and the onset of obesity.

### Circadian time point conditions

Mice were randomly assigned to light or dark cycle perfusion groups. One hour before the light cycle [zeitgeber time 23 (ZT23)] and 4 h before the dark cycle (ZT8), mice were anesthetized and perfused under their respective dark/light conditions.

### MCHR1 antagonist treatment

As previously described, mice were given an injection of the MCHR1 antagonist GW803430 (GW; 3 mg/kg, i.p.; catalog #4242, Tocris Bioscience) or vehicle control for 7 d, 3 h after the start of the light cycle (Alhassen et al., 2022). One week before the start of injections, mice were singly housed. Body weights were measured on the first day before injections to calculate the correct vehicle volume and dosage of GW treatment. MCHR1 antagonist was made fresh daily at a concentration of 0.5 mg/ml in 2 ml aliquots, (1 mg of GW, 8 µl of acetic acid, 1.6 ml of water, 125 µl of 2% Tween 80, and 100 µl of 1N NaOH). Mice were weighed on the morning of the last treatment day (day 7) and perfused 60–90 min after the last injection.

### Fixation and tissue processing

Mice were anesthetized with a 0.1 ml/10 g body weight dose of 2.0% tribromoethanol (Sigma-Aldrich) and transcardially perfused with PBS, followed by 4% paraformaldehyde in PBS (catalog #15710, Electron Microscopy Sciences). Brains were postfixed in 4% paraformaldehyde for 4 h at 4°C and then cryoprotected using 30% sucrose in PBS for 16–24 h. Cryoprotected brains were embedded in optimal cutting temperature compound (catalog #4585, Thermo Fisher Scientific) and sectioned at 15 µm.

### Immunofluorescence

Sections were washed with PBS for 5 min, then permeabilized and blocked in a PBS solution containing 1% BSA, 0.3% Triton X-100, 2% (v/v) donkey serum, and 0.02% sodium azide for 30 min at room temperature. Sections were incubated with primary antibodies in blocking solution overnight at 4°C. Primary antibodies include anti-MCHR1 (rabbit pAB; 1:250 dilution; catalog #711649, Thermo Fisher Scientific), anti-adenylate cyclase 3 [ACIII; 1:1000 dilution; chicken polyclonal antibody (pAb); CPCA-ACIII, Encor], anti-mCherry (chicken pAb; 1:1000 dilution; catalog NBP2-25158, Novus), anti-MCH (1:200 dilution; rabbit mAb; catalog #274415, Abcam). Sections were then washed with PBS before incubating with secondary antibodies for 1 h at room temperature. Secondary antibodies include donkey conjugated Alexa Fluor 647 and 488 (1:1000; Thermo Fisher Scientific) against appropriate species according to the corresponding primary. All primary and secondary solutions were made in the blocking solution described above. Slides were then washed in PBS and stained with Hoechst nuclear stain (catalog #H3570, Thermo Fisher Scientific) for 5 min at room temperature. Coverslips were mounted using SlowFade Diamond Antifade Mountant (catalog #S36972, Thermo Fisher Scientific).

### Mchr1 antibody validation

**Figure 1.**
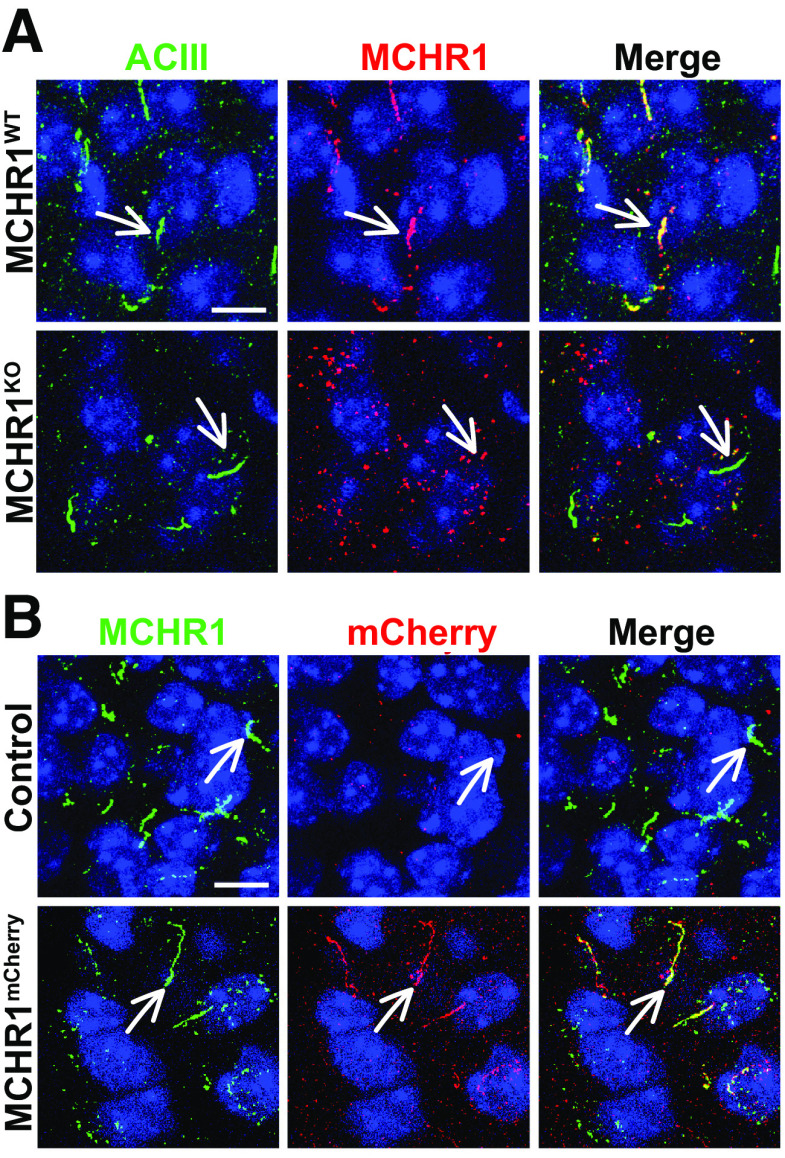
Antibody validation in MCHR1^KO^ and Mchr1^mCherry^ fusion allele animals. ***A***, MCHR1 knock-out mice show ACIII-positive cilia but show no MCHR1-positive cilia. ***B***, MCHR1 mCherry-tagged mice show colocalization of MCHR1 and mCherry tag-positive cilia. Scale bars, 10 µm. Hoechst nuclei blue stain was used. Arrows indicate example cilia. *N* = 3 animals/genotype.

Brain sections from previously described Mchr1 knock-out mice (*Mchr1^KO^*) and fluorescent reporter mice (*Mchr1^mCherry^*) were used for immunofluorescence to confirm the fidelity of the anti-MCHR1 antibody used throughout (Fig. 1*A*,*B*; Jasso et al., 2021).

### Confocal imaging

All images were acquired using a Leica SP8 confocal microscope in resonant scanning mode using a 63×, numerical aperture 1.4 objective. For all images collected, 16 bit image files were used for subsequent analysis.

### Image analysis

Cilia analysis was performed as previously described (Bansal et al., 2021). Briefly, sum projection images from captured *z*-stacks were analyzed using the artificial intelligence module, which had been trained to recognize cilia in brain section images. As part of the GA3 recipe, objects <1 μm in length were removed from the analysis. There were four to five mice per experimental condition, with four images captured per brain nucleus.

### Statistical analysis

All statistical tests were performed using GraphPad Prism. All statistically significant observations are noted in the figures and specific tests used are named within the legends.

## Results

To understand whether cilia GPCRs dynamically localize *in vivo* under physiological contexts associated with receptor activity, we initially chose to assess the known ciliary GPCR MCHR1. We assessed its ciliary localization in conjunction with the broadly expressed CNS ciliary membrane-associated ACIII (Bishop et al., 2007; Berbari et al., 2008b; Hsiao et al., 2021; Kobayashi et al., 2021; Alhassen et al., 2022). We confirmed our MCHR1 antibody immunofluorescence specificity by observing the loss of ciliary staining in a *Mchr1* knock-out allele mouse brain and colocalization with a *Mchr1-mCherry* knock-in fusion allele mouse (Fig. 1; Jasso et al., 2021). For our broader analysis of cilia localization, we used our recently reported computer-assisted approach for measuring cilia frequency, length, and fluorescence intensity (Bansal et al., 2021). This approach offers the advantages of being less biased and having higher throughput.

**Figure 2.**
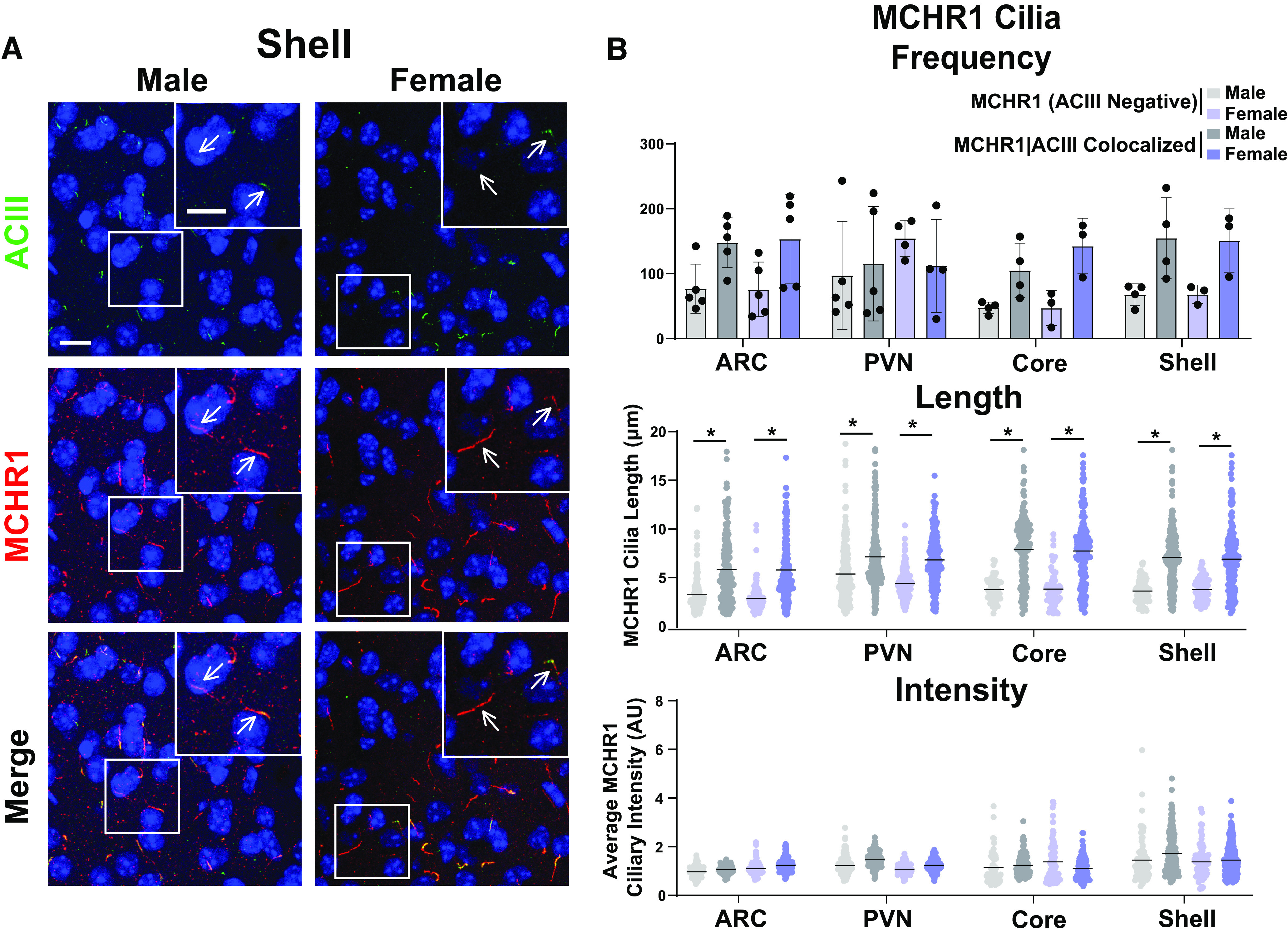
MCHR1 cilia localization is similar in adult male and female mice. ***A***, Representative immunofluorescence images of neuronal cilia (ACIII, green) and MCHR1 (red) in the Shell of males and females. Scale bars, 10 µm. Hoechst nuclei blue stain was used. Arrows indicate example cilia. ***B***, Mean MCHR1 cilia frequency per animal in the ARC, PVN, and the core and shell of the nucleus accumbens for cilia that have only MCHR1 [MCHR1 (ACIII Negative)] and cilia that have both MCHR1 and ACIII (MCHR1|ACIII Colocalized). Mean MCHR1 cilia length and intensity in MCHR1 (ACIII Negative) cilia or in MCHR1|ACIII colocalized cilia in the ARC, PVN, and the core and shell (nested *t* test, *p* > 0.05 for all male vs female comparisons in each region). *N* = 5 animals/group with an average of 250 cilia per brain nuclei of each animal analyzed. *p < 0.05.

As the MCH and MCHR1 signaling axis displays sexual dimorphism, our initial analysis compared cilia frequency, length, and fluorescence intensity in adult male and female mice (Messina et al., 2006; Santollo and Eckel, 2008). Surprisingly, we did not observe differences in cilia frequency, length, or MCHR1 intensity in any of the brain regions assessed, including the hypothalamic arcuate (ARC) and paraventricular nucleus (PVN), and the nucleus accumbens (shell and core) between males and females (Fig. 2). Interestingly, we did observe length differences between MCHR1-only, (ACIII negative) positive cilia and MCHR1|ACIII double-positive cilia, where MCHR1|ACIII colocalized cilia were significantly longer (Fig. 2*B*). This length difference between the two cilia populations was observed throughout our data. As we did not observe differences between males and females, we continued the remaining studies using adult males.

**Figure 3.**
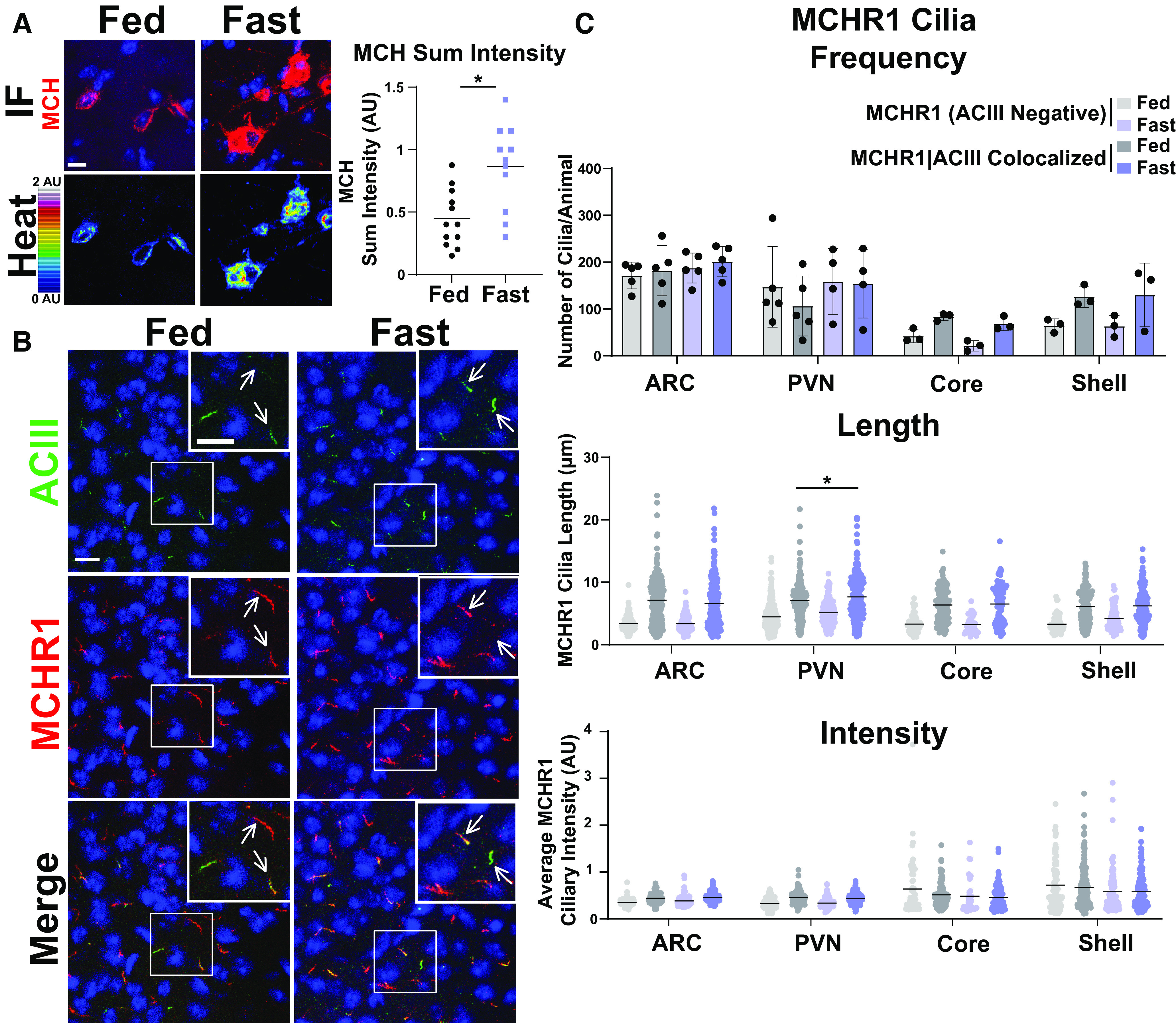
Acute feeding status alters MCHR1 length specifically in the PVN. ***A***, MCH immunofluorescence staining (red) and intensity measurement (Heat) significantly increased under fasted conditions in the lateral hypothalamus (Student’s *t* test, *p* = 0.0024, 0.415 ± 0.120 a.u.). ***B***, Representative immunofluorescence images of neuronal cilia (ACIII, green) and MCHR1 (red) in the PVN of *ad libitum*-fed (Fed) and fasted (Fast) animals. Scale bars, 10 µm. Hoechst nuclei blue stain was used. Arrows indicate example cilia. ***C***, Mean MCHR1 cilia frequency per animal in the ARC, PVN, and the core and shell of the nucleus accumbens for cilia that have only MCHR1 [MCHR1 (ACIII Negative)] and cilia that have both MCHR1 and ACIII (MCHR1|ACIII Colocalized). Mean MCHR1 cilia length and intensity in cilia with just MCHR1 (ACIII Negative) or in MCHR1|ACIII colocalized cilia. Significant changes in MCHR1|ACIII colocalized cilia length were observed in the PVN (nested *t* test, *p* = 0.020, 0.62 ± 0.21 μm). *N* = 5 animals/treatment group with an average of 200 cilia/brain nucleus of each analyzed. *p < 0.05.

**Figure 4.**
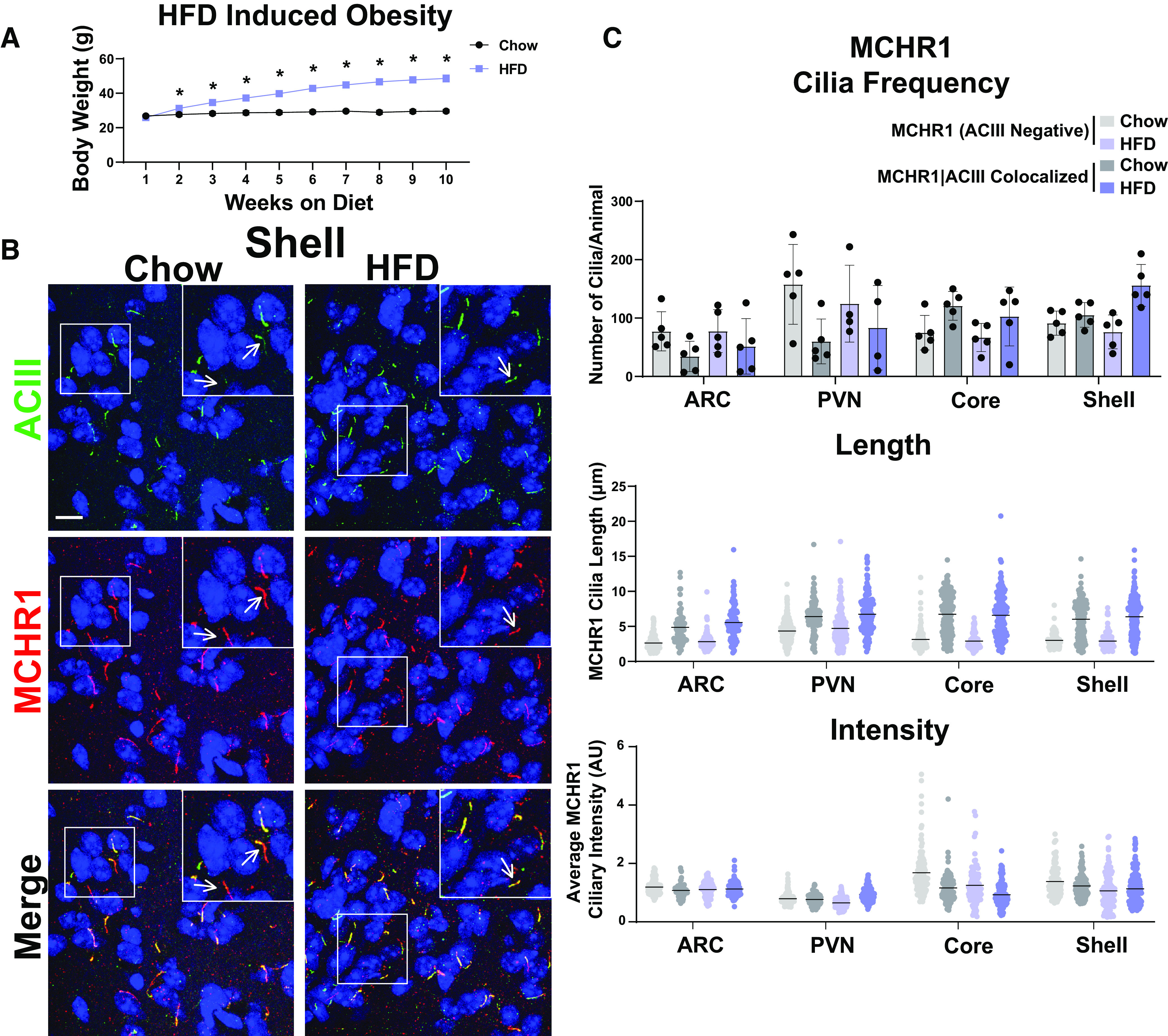
HFD-induced obesity does not influence MCHR1 cilia localization. ***A***, High-fat diet-induced obese and chow-fed control animal body weights (Student’s *t* test; *p* = 0.008 at 2 weeks and is <0.0001 onward). ***B***, Representative immunofluorescence images of neuronal cilia (ACIII, green) and MCHR1 (red) in the Shell of control diet (Chow) and HFD-induced obese males. Scale bars, 10 µm. Hoechst nuclei blue stain was used. Arrows indicate example cilia. ***C***, Mean MCHR1 cilia frequency per animal in the ARC, PVN, and the core and shell of the nucleus accumbens for cilia that have only MCHR1 [MCHR1 (ACIII Negative)] and cilia that have both MCHR1 and ACIII (MCHR1|ACIII Colocalized). Mean MCHR1 cilia length and intensity in cilia with just MCHR1 [MCHR1 (ACIII Negative)] or in MCRH1|ACIII colocalized cilia (nested *t* test, *p* > 0.05). *N* = 5 animals per treatment group with an average of 250 cilia/animal and nuclei analyzed. *p < 0.05.

**Figure 5.**
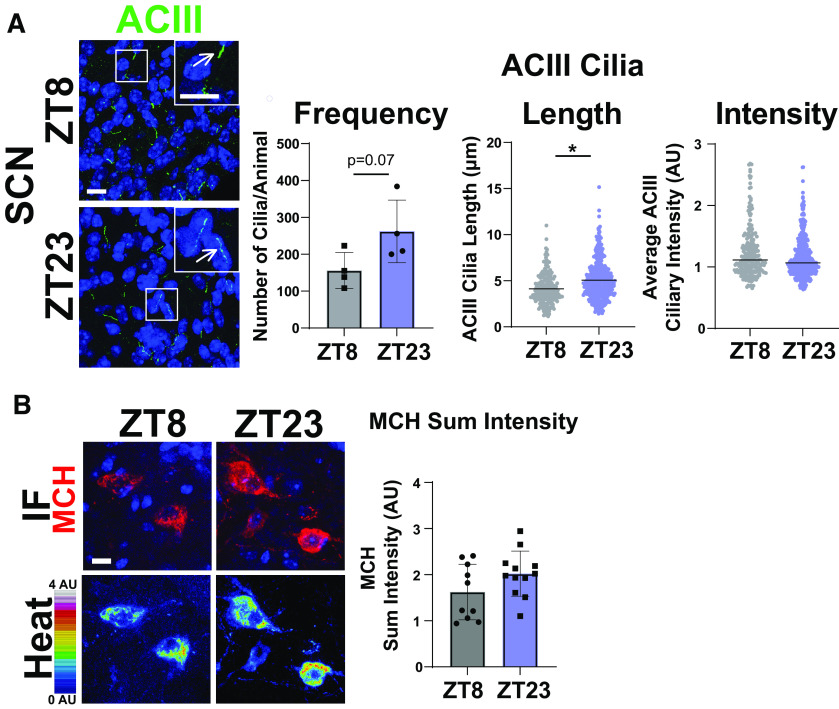
ACIII ciliary localization is altered at ZT23, while MCH levels do not change. ***A***, SCN ACIII cilia length at ZT23 (dark cycle; nested *t* test, *p* = 0.0291, 0.74 ± 0.26 μm). ***B***, MCH immunofluorescence staining (red) and intensity measurement (Heat) is not significantly different in the lateral hypothalamus between ZT8 and ZT23. *p < 0.05.

**Figure 6.**
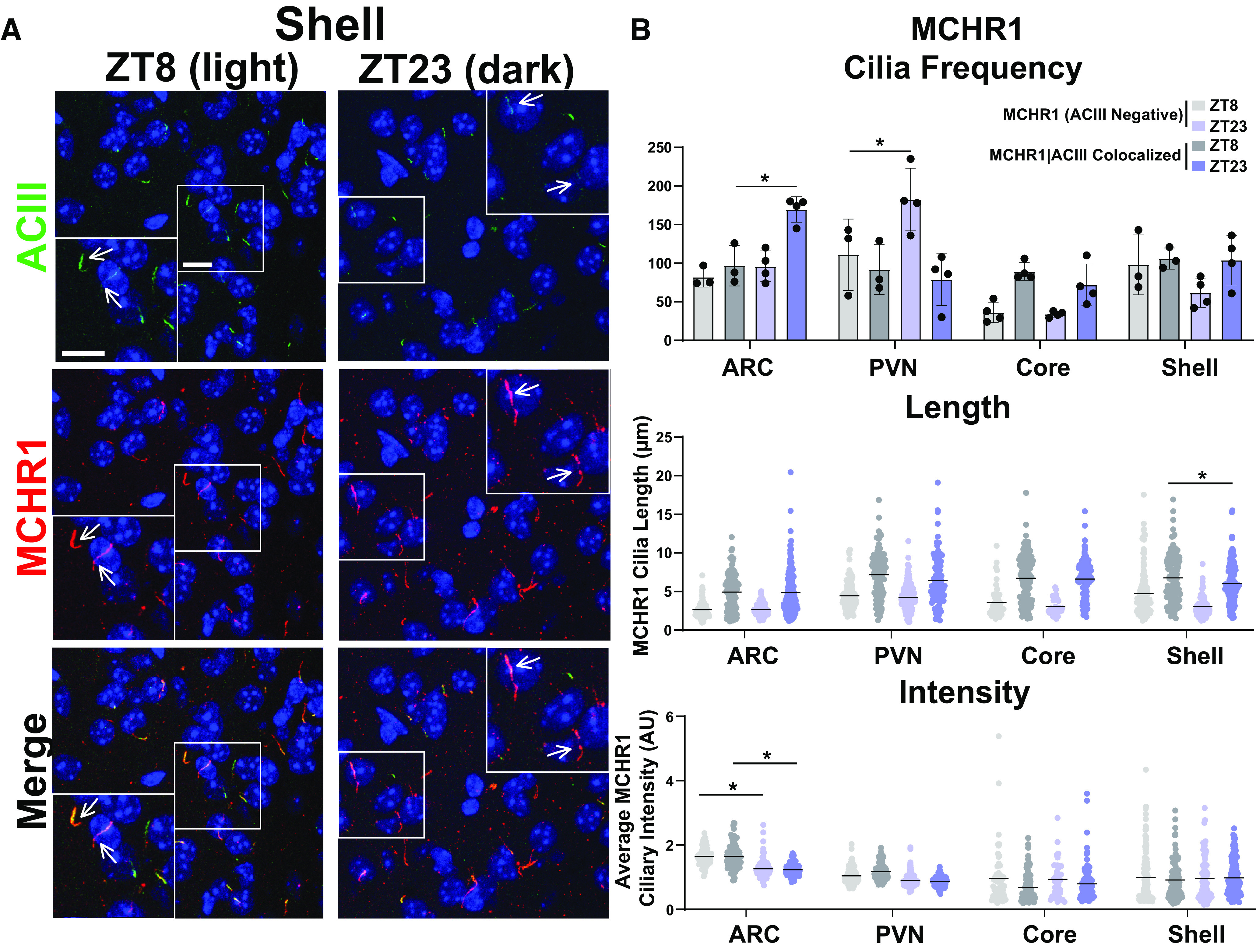
MCHR1 cilia localization is influenced by circadian rhythm. ***A***, Representative immunofluorescence images of neuronal cilia (ACIII, green) and MCHR1 (red) in the shell at ZT8 (light cycle) and ZT23 (dark cycle) timepoints. Scale bars, 10 µm. Hoechst nuclei blue stain was used. Arrows indicate example cilia. ***B***, MCHR1 cilia frequency per animal in the ARC, PVN, Core, and Shell at ZT8 and ZT23 for cilia that have only MCHR1 [MCHR1 (ACIII Negative)] and cilia that have both MCHR1 and ACIII (MCHR1|ACIII Colocalized; two-way ANOVA; ARC: *p* = 0.004, 73 ± 20 cilia; PVN: *p* = 0.005, 70 ± 20 cilia). Mean MCHR1 cilia length and intensity in MCHR1 (ACIII Negative) and MCHR1|ACIII colocalized cilia. Significant decreases in MCHR1 cilia length in MCHR1|ACIII cilia in the shell and significant decreases in MCHR1 (ACIII Negative) cilia fluorescence intensity in the ARC at ZT23 (nested *t* test; accumbens shell: *p* = 0.0089, −0.94 ± 0.23 µm; ARC: *p* = 0.0168, 0.386 ± 1.10 a.u.; *p* = 0.0147, −0.454 ± 1.24 a.u., respectively). *N* = 5 and 4 animals/treatment group, respectively, with an average of 200 cilia/animal and nuclei analyzed. *p < 0.05, **p < 0.01.

MCHR1 function has been extensively implicated in feeding behaviors, body weight, and energy homeostasis (for recent review, see Al-Massadi et al., 2021). Its ligand, MCH, is increased following acute fasting (Simon et al., 2018). Upon a 16 h fast, we observed an increase in MCH ligand immunostaining in the lateral hypothalamus, the known site of MCH expression (Fig. 3*A*; Zamir et al., 1986). We next assessed the impact of fasting on ciliary MCHR1 in hypothalamic nuclei associated with this behavior, the ARC and PVN, and the nucleus accumbens, a site of high MCHR1 ciliary localization (Berbari et al., 2008a). We did not observe changes in cilia frequency or MCHR1 intensity (Fig. 3*B*,*C*). Surprisingly, we only observed significant fasting-associated increases in MCHR1|ACIII colocalized cilia length within the PVN (Fig. 3*C*). To determine whether body weight and obesity can influence MCHR1 ciliary localization, we assessed the brains of high-fat diet-induced obese mice (Fig. 4*A*). Obesity did not influence cilia frequency, length, or MCHR1 fluorescence intensity in the ARC, PVN, or accumbens (Fig. 4*B*,*C*). MCHR1 signaling has also been implicated in sleep/wake cycles (Blanco-Centurion et al., 2019). To determine whether MCHR1 cilia localization changes with the light cycle, we assessed brains at ZT8 (light) and ZT23 (dark). We initially assessed the suprachiasmatic nucleus (SCN), the classic region involved in circadian rhythms and where light cycle-associated cilia length changes have recently been implicated (Hastings et al., 2018; Tu et al., 2022). While we do not observe MCHR1-positive cilia in the SCN, we did note changes in ACIII cilia similar to those observed by Tu et al. (2022; Fig. 5*A*). Staining for the MCH ligand at both ZT8 and ZT23 did not show changes (Fig. 5*B*). Interestingly, we also observed changes in MCHR1 cilia frequency in the ARC and PVN during the light/dark cycle with more cilia being observed in the dark (ZT23; Fig. 6*A*,*B*). In addition, MCHR1|ACIII colocalized cilia length in the shell of the accumbens appeared shorter in the dark cycle (ZT23; Fig. 6*B*). In the ARC, the average MCHR1 fluorescence intensity was significantly reduced in both populations of cilia at ZT23 (Fig. 6*B*).

**Figure 7.**
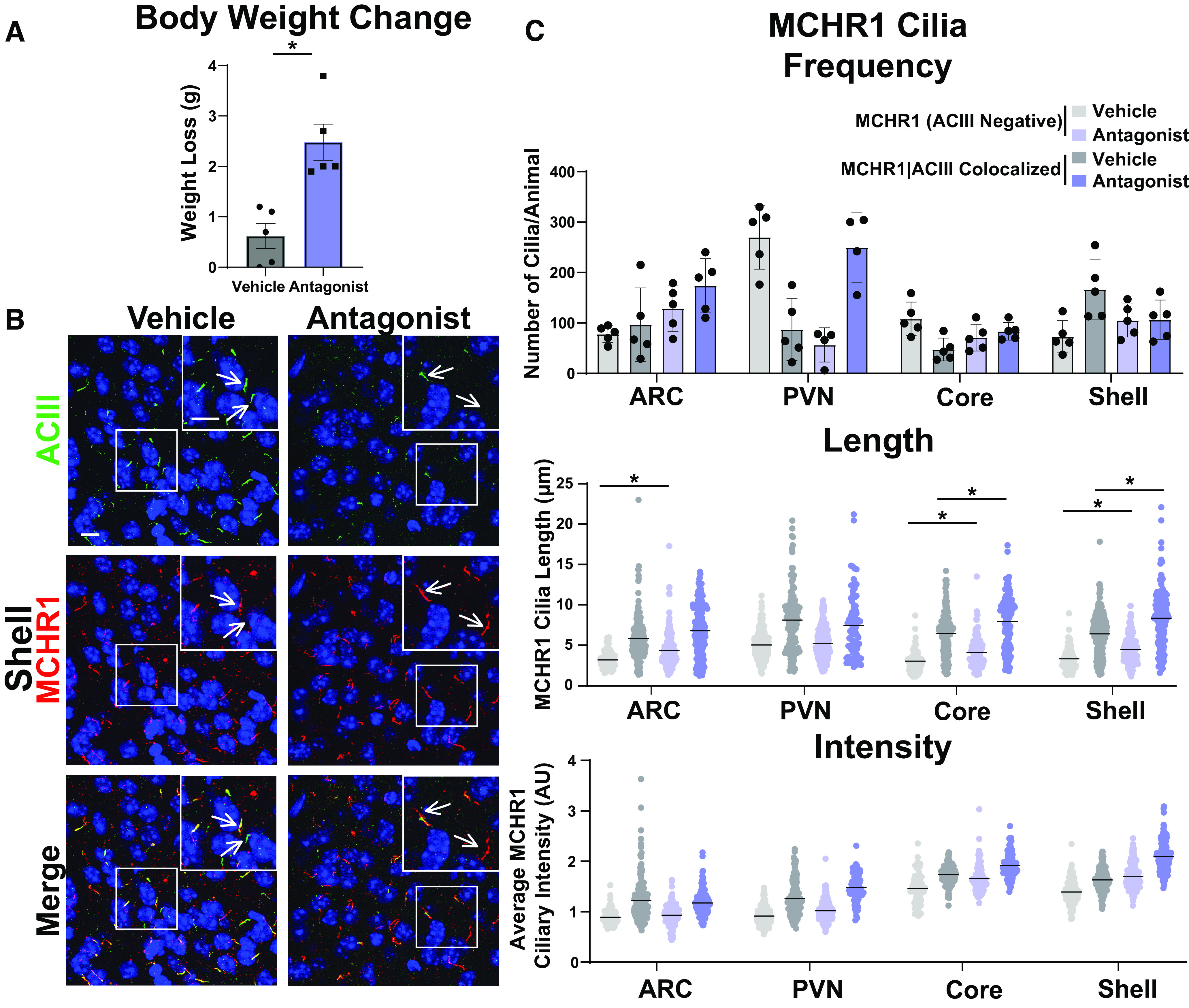
Antagonism alters MCHR1 length in the ARC and NA. ***A***, Antagonist treatment causes significant weight loss (Student’s *t* test; *p* = 0.002, 1.860 ± 0.4368 g). ***B***, Representative immunofluorescence images of neuronal cilia (ACIII, green) and MCHR1 (red) in the shell of control animals (Vehicle) and MCHR1 antagonist-treated animals (Antagonist). Scale bars, 10 µm. Hoechst nuclei blue stain was used. Arrows indicate example cilia. ***C***, MCHR1 cilia frequency in the ARC, PVN, Core, and Shell after vehicle and antagonist treatment. Significant increase in MCHR1 only cilia [MCHR1 (ACIII Negative)] after antagonist treatment in the ARC (two-way ANOVA; *p* = 0.008, 96 ± 28 cilia). Mean MCHR1 cilia length and fluorescence intensity in cilia with MCHR1 (ACIII Negative) and with MCHR1|ACIII-colocalized cilia. Significant changes in cilia length for both cilia populations in the ARC, Core, and Shell [MCHR1 (ACIII Negative) nested *t* test; ARC: *p* = 0.0089, 0.94 ± 0.23 µm; accumbens core: *p* = 0.0033, 0.97 ± 0.31 µm; accumbens shell: *p* = 0.0224, 0.89 ± 0.31 µm; MCRH1|ACIII Colocalized cilia: nested *t* test; accumbens core: *p* = 0.0003, 1.47 ± 0.24 µm; accumbens shell: *p* < 0.0001, 1.70 ± 0.22 µm, respectively]. *N* = 5 animals/treatment group with an average of 250 cilia/animal and nuclei analyzed. *p < 0.05, **p < 0.01.

After assessing multiple physiological conditions where MCHR1 function has been implicated, we next looked to see whether overt pharmacological antagonism could influence MCHR1 ciliary localization. Injection of the antagonist GW803430 for 7 d resulted in significant loss in body weight (Fig. 7*A*; Alhassen et al., 2022). MCHR1 antagonism increased the frequency of MCHR1|ACIII colocalized cilia in the ARC and in the PVN (Fig. 7*C*). Antagonism also increased ciliary length in the accumbens core and shell for both cilia populations (Fig. 7*C*). Interestingly, in the ARC cilia length increases were observed only in MCRH1 (ACIII negative) cilia (Fig. 7*C*).

**Figure 8.**
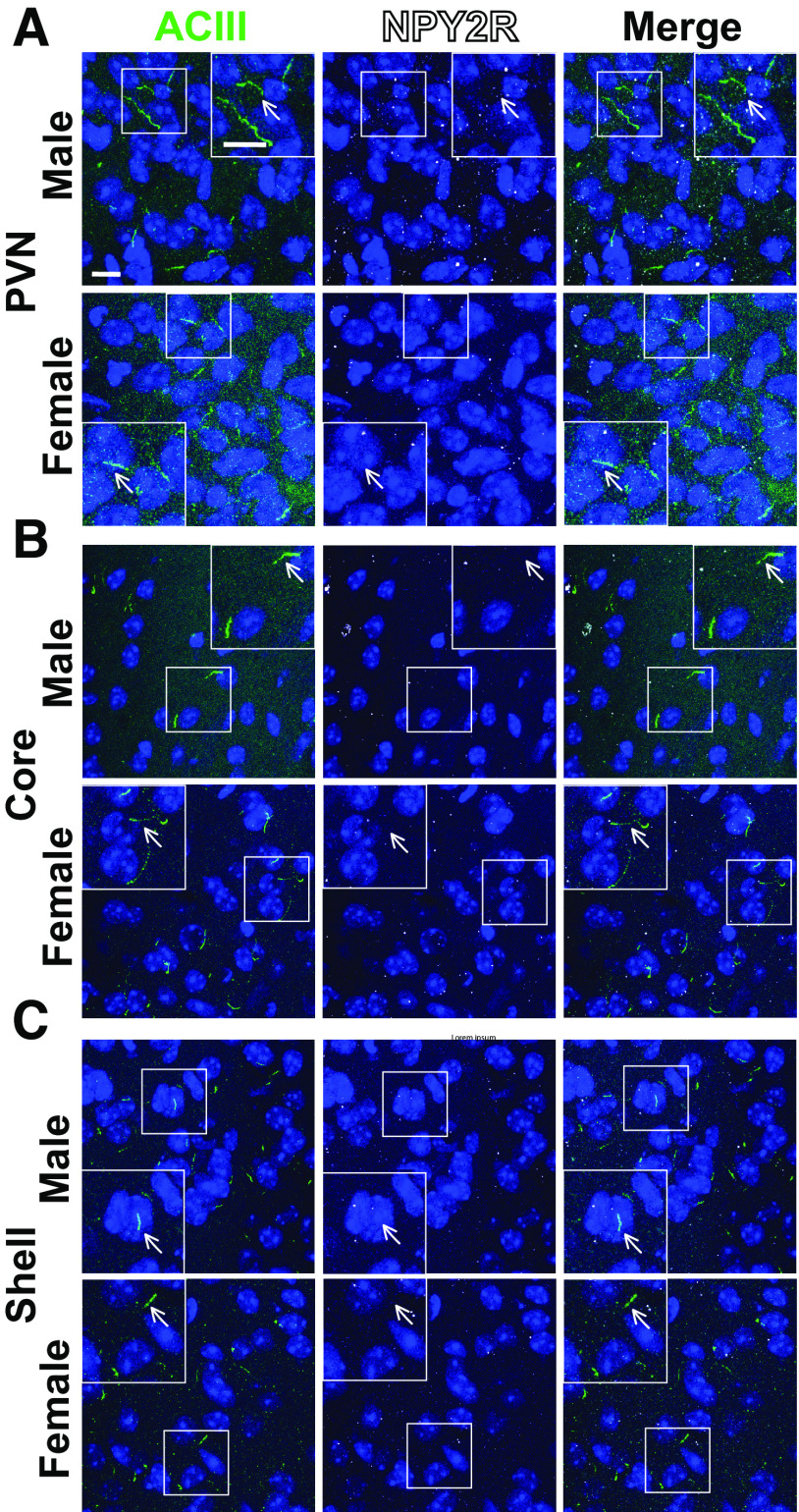
NPY2R does not localize to cilia in the PVN or nucleus accumbens. ***A–C***, Representative immunofluorescence images of neuronal cilia (ACIII, green) and NPY2R (white) within the PVN (***A***), nucleus accumbens core (***B***), and nucleus accumbens shell (***C***). Scale bars, 10 µm. Hoechst nuclei blue stain was used. Arrows indicate example cilia.

**Figure 9.**
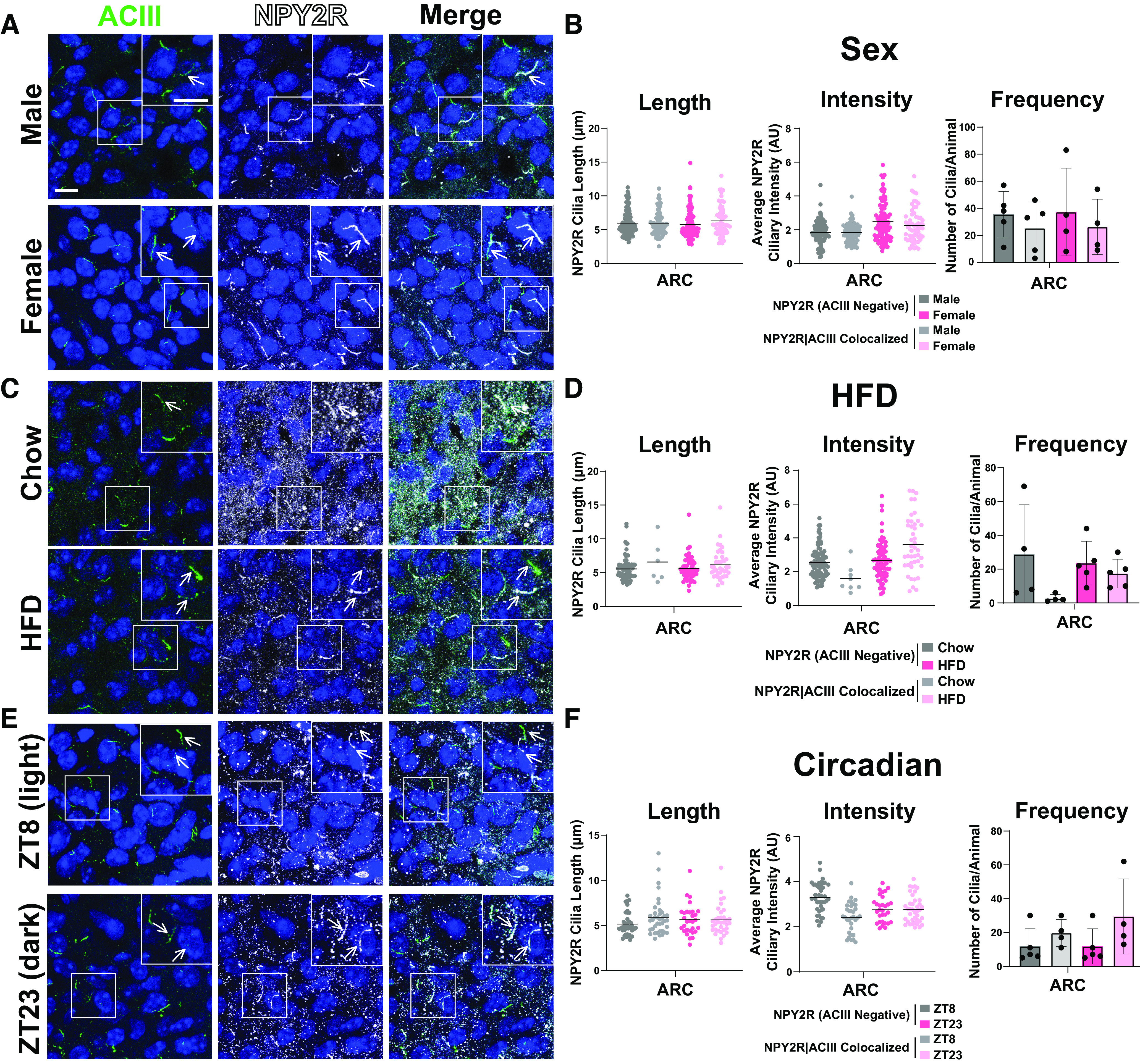
NPY2R cilia localization in the ARC is unchanged among sexes, obesity, and circadian times. ***A, C, E***, Representative immunofluorescence images of neuronal cilia (ACIII, green) and NPY2R (white) within the ARC between male and female, HFD obese and control chow, and at ZT8 (light) and ZT23 (dark). Scale bars, 10 µm. Hoechst nuclei blue stain was used. Arrows indicate example cilia. ***B, D, F,*** Mean NPY2R cilia frequency per animal for cilia that have only NPY2R [NPY2R (ACIII Negative)] and cilia that have both NPY2R and ACIII (NPY2R|ACIII Colocalized). Mean NPY2R cilia length and intensity in NPY2R (ACIII Negative) cilia or in NPY2R|ACIII colocalized cilia in the ARC under the conditions (Sex, HFD, Circadian).

**Figure 10.**
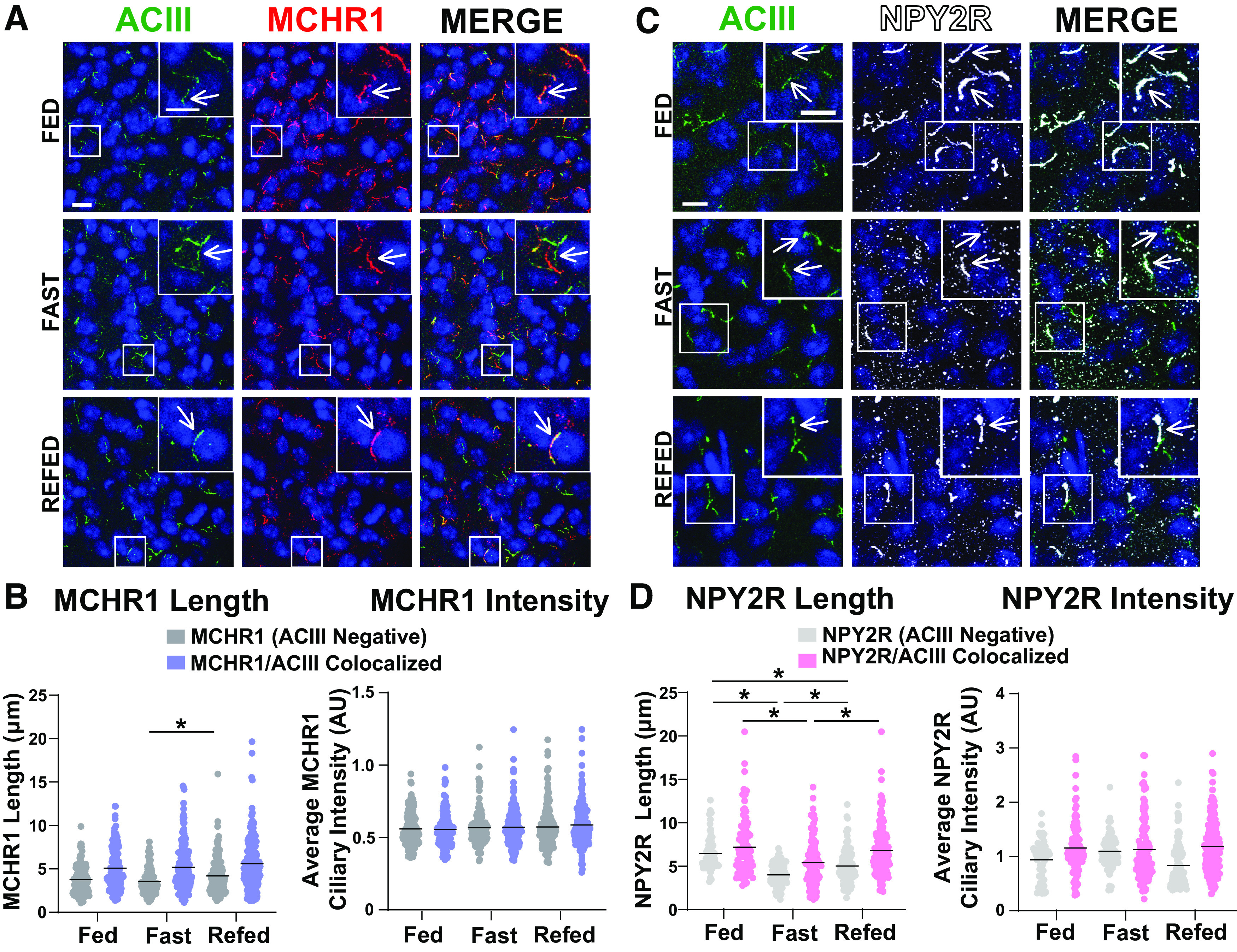
NPY2R changes under different feeding conditions in the ARC. ***A***, Representative immunofluorescence images of neuronal cilia (ACIII, green) and MCHR1 (red) in the ARC of *ad libitum*-fed (Fed), overnight fasted (Fast), and 4 h postrefeeding after fast (Refed) conditions. Scale bars, 10 µm. Hoechst nuclei blue was used. Arrows indicate example cilia. ***B***, Mean MCHR1 cilia length and intensity in cilia that have only MCHR1 [MCHR1 (ACIII Negative)] and in cilia with both MCHR1 and ACIII (MCRH1|ACIII Colocalized Cilia) in Fed, Fast, and Refed animals. Significant increase in MCHR1 (ACIII Negative) cilia length on refeeding (nested one-way ANOVA: *p* = 0.004, 0.654 ± 0.201 µm). ***C***, Representative immunofluorescence images of neuronal cilia (ACIII, green) and NPY2R (white) in the ARC of Fed, Fast, and Refed conditions. ***D***, Mean NPY2R cilia length and intensity in cilia with only NPY2R [NPY2R (ACIII Negative)] or in cilia with both NPY2R and ACIII (NPY2R|ACIII Colocalized) in Fed, Fast, and Refed animals. NPY2R (ACIII negative) cilia in the ARC significantly change length on Fed, Fast, and Refed conditions (nested one-way ANOVA, *p* < 0.001, −2.47 ± 0.29 µm; *p* < 0.00011.45 ± 0.28 µm; *p* = 0.0002, −1.02 ± 0.25 µm, respectively). NPY2R|ACIII colocalized cilia are also significantly shorter on fasting and remain slightly shorter in the Refed condition compared with Fed (nested *t* test, *p* < 0.0001, −1.80 ± 0.41 µm; *p* = 0.0003, 1.43 ± 0.37 µm, respectively). Each data point represents a cilium. Scale bars, 10 µm. Hoechst nuclei blue stain was used. *N* = 5 animals/group with an average of 250 cilia/animal. *p < 0.05.

To determine whether these results are specific to MCHR1 or perhaps applicable to multiple neuronal ciliary GPCRs, we assessed the localization of NPY2R, another GPCR known to localize to cilia (Loktev and Jackson, 2013). We focused our analysis on the ARC as we did not observe NPY2R cilia localization in other brain regions of interest in males or females (Fig. 8). Within the ARC, we also did not observe changes in NPY2R cilia between sexes, in HFD-induced obesity or at different circadian times (Fig. 9). Similar to MCH, acute fasting also increases the levels of the NPY2R ligand NPY (Yasrebi et al., 2016). Thus, we sought to assess both MCHR1 and NPY2R on fasting and refed states (Fig. 10). We only observed significantly longer MCHR1 (ACIII-negative) cilia lengths in the refed condition compared with the fasted (Fig. 10*A*,*B*). However, we observed significant cilia length changes in NPY2R (ACIII-negative) and NPY2R|ACIII colocalized cilia. NPY2R cilia were significantly longer in both the *ad libitum* fed and refed conditions compared with the fasted condition (Fig. 10*C*,*D*). These results demonstrate that dynamic localization to cilia is dependent on properties of the individual receptor and the brain region of expression *in vivo.*

## Discussion

Cilia are recognized as mediators of diverse signaling pathways, yet many questions remain unanswered regarding how they coordinate signaling. In cell line and heterologous expression systems *in vitro*, dynamic localization of receptors to the cilia membrane has been reported for a number of ciliary GPCRs, including MCHR1 (Ye et al., 2018). *In vivo* dynamic localization to the cilia as a means of signaling control has been best described for cilia-mediated hedgehog signaling during development (Bangs and Anderson, 2017). We sought to determine whether cilia broadly deploy dynamic GPCR localization *in vivo* to mediate signaling. We chose a ciliary receptor associated with several physiological states and phenotypes, including sexual dimorphic expression, acute feeding behavior, energy homeostasis, and sleep (Al-Massadi et al., 2021). MCHR1 also has the advantage of being the only known receptor for MCH in mice (Diniz and Bittencourt, 2019). In contrast, many other ciliary GPCRs are within a family of receptors for certain neuropeptides. For example, the ciliary somatostatin receptor 3 (SSTR3) is one of five receptors (SSTR1-5) for the ligand somatostatin (Yamada et al., 1992a, b, 1993).

Our initial assessment of MCHR1 focused on the hypothalamus for a number of reasons. Ciliopathies are known to have deficits in hypothalamic control of energy homeostasis (Davenport et al., 2007; Sun et al., 2021; Wang et al., 2021c). MCHR1 fails to localize properly in obese ciliopathy models of Bardet–Biedl syndrome (BBS; Berbari et al., 2008a). In addition, *Mchr1* expression is observed in several hypothalamic nuclei under baseline conditions (Engle et al., 2018). MCHR1 signaling has also been extensively implicated in feeding behavior, energy homeostasis, and metabolism. Agonism or activation of the pathway is associated with increases in food intake, and loss-of-function alleles or pharmacological antagonism associated with weight loss (for recent review of MCH and MCHR1 signaling, see Al-Massadi et al., 2021).

We chose an antibody staining approach combined with a computer-assisted analysis as this combination was the best way to detect endogenous ciliary MCHR1 in an unbiased and high-throughput manner. It also allows us to readily observe hundreds of cilia per animal (Bansal et al., 2021; Jasso et al., 2021).

We were surprised to find that our analysis revealed that MCHR1 ciliary localization remained largely fixed across males and females, on fasting and diet-induced obesity, with only subtle significant changes observed in cilia length. We also observed that MCHR1|ACIII-colocalized cilia were significantly longer than MCHR1 only (ACIII negative) cilia. Our observation that ACIII cilia length changes within the SCN depending on the light or dark cycle as recently reported in a preprint (Tu et al., 2022), assured us that our analysis could detect broad-scale changes in cilia lengths, frequency, and localization. It was interesting that we also detected length decreases in MCHR1|ACIII colocalized cilia in the shell of the nucleus accumbens in the dark cycle (Becker-Krail et al., 2022). This suggests the potential for cilia-mediated signaling changes broadly in the brain based on light conditions.

Pharmacological MCHR1 antagonism demonstrated the most substantial changes in both cilia length and intensity across different brain regions, but this approach may not be physiologically relevant. However, this result is in line with what cilia have been proposed to do when their GPCR-associated signaling system is saturated or overwhelmed by changing their lengths and shedding cilia-specific vesicles (Nager et al., 2017; Phua et al., 2017). These phenomena have been directly observed for cilia in BBS cell models (Nager et al., 2017). It remains to be seen how common cilia length regulation and vesicular shedding is deployed as a means of cilia-mediated signaling *in vivo*. It is possible that both are important processes, but that under normal physiological conditions they remain challenging to detect in mammalian systems *in vivo* with currently available tools.

To further explore the possibility that other cilia GPCRs could be relatively stationary *in vivo*, we investigated another hypothalamic ciliary GPCR under physiological conditions in which it has been implicated: NPY2R and feeding status (Loktev and Jackson, 2013). Interestingly, for NPY2R, we observed significant changes in length for both cilia populations with fasted cilia being shorter and refed cilia being longer compared with *ad libitum*-fed animals. These data suggest that NPY2R cilia are more dynamic on acute changes in feeding when compared with MCHR1 cilia. At the neuroanatomical level, our data reveal that specific brain regions independently localize certain receptors to their cilia. In other words, the MCHR1/MCH signaling axis localization behaves differently dependent on the anatomic context. This opens up the possibility that ciliary GPCRs may be dynamic depending on what tissue is being investigated. For example, MCHR1 is potentially expressed in peripheral tissues, and its ciliary localization in these contexts is unclear (Balber et al., 2019). Overall, these data further point to the potential that many ciliary GPCRs may need to be assessed independently and in tissues and cells of interest to learn how their signaling is mediated *in vivo*.

At the receptor level, our data point to the potential for specific G-protein coupling being important for dynamic localization to cilia. MCHR1 is thought to be Gα**_i_** coupled while NPY2R is Gα**_s_** coupled. However, coupling at the cilia for most nonodorant ciliary GPCRs is undetermined (Loktev and Jackson, 2013; Saito et al., 2013). Our data also may reflect the inherent nature of some GPCRs being more dynamic at membranes compared with others (Schmidt et al., 2014). It is also possible that in some cases the pool of receptors that is critical for signaling is on the plasma membrane and not the ciliary membrane, and thus cilia localization appears stable for a given GPCR. Future studies will assess how G-protein coupling and other pools of receptors may specifically influence ciliary GPCR localization. For example, Gα**_s_** (e.g., NPY2R) ciliary receptors may be generally more dynamic to the compartment compared with those that couple to other Gα subunits (e.g., MCHR1).

Together our results demonstrate that dynamic localization to the ciliary compartment may not apply to some physiological conditions *in vivo* or be a common theme across ciliary GPCRs. Our results also suggest that only specific ciliary GPCRs use length control as a mechanism to mediate signaling, as may be the case for NPY2R but not MCHR1. Finally, our results also demonstrate that localization across different brain regions and nuclei that all possess the same ciliary GPCR are dynamically regulated differentially. For example, even on supraphysiological antagonism of MCHR1, we did not observe the same changes in cilia length and localization in all brain regions analyzed. Ultimately, a comprehensive understanding of how cilia mediate GPCR signaling could provide therapeutic opportunities for cilia-receptor ligands in conditions like obesity.

## Synthesis

Reviewing Editor: Masha Prager-Khoutorsky, McGill University Decisions are customarily a result of the Reviewing Editor and the peer reviewers coming together and discussing their recommendations until a consensus is reached. When revisions are invited, a fact-based synthesis statement explaining their decision and outlining what is needed to prepare a revision will be listed below. The following reviewer(s) agreed to reveal their identity: Melissa Chee. Note: If this manuscript was transferred from JNeurosci and a decision was made to accept the manuscript without peer review, a brief statement to this effect will instead be what is listed below.

appended below

## Author Response

We thank the reviewers for their feedback. Our updated manuscript attempts to address all of the critiques with editorial changes, revised figures, and additional data. We believe these changes improve the manuscript. Our responses to reviewer comments follow.

The writing of the paper could use improvement, particularly from an organizational standpoint. It was unclear from the abstract and even the introduction exactly what I was going to learn from this paper. For example, is this paper about the trafficking of specific GPCRs to and from the cilia in response to physiological conditions, or about physical regulation of cilia by these pathways in response to physiological conditions, or about cilia heterogeneity? Or maybe about each of these things? Some further clarity or perhaps a clear hypothesis would help this.

We appreciate this suggestion. While our work addresses aspects of all these questions, we have elected to focus on a couple of them in the abstract and introduction and leave the other parts for the discussion. For example, we now focus on GPCR dynamic localization to cilia in vivo to launch the results section. We offer a revised abstract and introduction to support this focus.

The abstract itself states that “no consistent theme” emerges from the study, which lessened my enthusiasm for the paper. This is an overly negative way of framing things right from the start, and is also ambiguous- what even were the themes being tested/examined?. I suspect this could be reframed with more careful consideration to what these findings *do* tell us, and by making more of an attempt to cohesively present this data.

We agree our framing needed improvement. We address this in the following ways:

Abstract: We now explicitly state, “These data suggest that dynamic cilia localization of

GPCRs may depend on properties of individual receptors.”

Introduction: “To determine if these GPCRs dynamically localize to cilia in vivo, we assessed their localization under different feeding conditions. We hypothesized that cilia GPCRs throughout the CNS would dynamically localize to the compartment based upon changes in signaling, similar to other model systems and heterologous cell line data.”

Results:

• We highlight two types of cilia throughout GPCR (ACIII negative) and GPCR|ACIII colocalized populations.

• We have incorporated more NPY2R data into new Figures 8, 9 and 10.

• Cilia frequency data is now throughout the figures as a separate graph.

• We now compare GPCR (ACIII Negative) and GPCR|ACIII Colocalized cilia population data across conditions within the same graphs.

• We explicitly indicate increases and decreases in frequency, length, and intensity. 2

Discussion:

• We have added additional rationale for why we initially focused on MCHR1. It is the only known receptor for the MCH system in mice, whereas most other ciliary GPCRs lie within a family of receptor isoforms (i.e., somatostatin receptors 1-5).

• We have added ideas as to why one GPCR may be more dynamic than the other. For example, perhaps G-protein coupling preference is associated with dynamic localization (i.e., MCHR1-Gαi is stable at cilia while NPY2R-Gαs is dynamic at cilia). The NPY2R data had not been fully considered and felt a bit tacked on at the end, especially given that it shows some interesting responses. The authors should look at NPY2R under similar conditions to MCHR1 and compare and contrast these two GPCRs more extensively.

We agree and have looked at NPY2R in different conditions and brain regions; we have added these data to new Figures 8 and 9.

The distinction between MCHR1 and MCHR1/ACIII cilia was also somewhat ambiguous. Are the MCHR1 alone cilia ACIII negative or was ACIII just not evaluated there? If there are these distinct populations of cilia, it might be worth commenting further on what may be different between these populations.

We apologize for our lack of clarity. In all contexts, staining was simultaneously performed for the GPCR of interest (MCHR1 or NPY2R) and ACIII. To clarify for the reader, we have revised our wording within the results and the labels on the figures and graphs. The terms MCHR1 (ACIII negative) and MCHR1|ACIII colocalized cilia are used throughout. We also highlight the differences between these cilia populations in revised Figure 2, where we observe length differences. MCHR1|ACIII colocalized cilia are longer across all experiments compared to MCHR1 (ACIII negative) cilia. We have also included this as a discussion point in the revised manuscript.

Ciliary MCHR expression was analyzed in MCHR1 and MCHR1+ACII cilia. This appears to be an important addition. However, please also provide direct comparisons between MCHR1 vs MCHR1+ACII cilia. It would also be helpful to indicate whether MCHR1 only cilia represent a unique subpopulation of cilia - what might be some other markers available to classify them.

We have added the requested analysis throughout. Unfortunately, the prototypical markers for assessing cilia do not work in the adult brain (i.e., acetylated α-tubulin). In addition, markers thought to be ubiquitous to cilia (i.e., ARL13b) do not appear so in the adult brain. Thus, our analysis is limited to known ciliary GPCRs with established antibodies and ACIII. We considered assessing cilia using a cilia-tagged allele, but felt that this approach would come with its own caveats, so we pursued assessing endogenous ciliary GPCRs using antibody staining with appropriate knockout and transgenic controls for MCHR1. 3

The authors should comment on differences in cilia parameters between MCHR1 cilia and MCHR1+ACII cilia? It appears from Figure 1 that MCHR1 cilia might be shorter than MCHR1+ACII cilia.

We now highlight the differences in revised Figure 2. The authors found that there were no sex differences in ciliary characteristics between cells from male vs female brains. This is an important analysis, but in addition to ciliary parameters, please also indicate if there were sex differences in the proportion of cells in the ARC, PVN, NAc - for example, is there differences in ciliary frequency.

We have provided cilia frequency data throughout the revised manuscript.

Is there a difference in the intensity of MCH expression at ZT8 and ZT23?

We have provided this analysis in new Figure 5B. We do not observe significant changes in MCH staining in the LH between ZT8 and ZT23.

There were a few instances in reporting the Results where the authors indicate there was a significant difference in XXX (e.g., XXX = MCHR1 cilia length in PVN; changes in cilia frequency in ARC and PVN; etc) but the Results do not report the direction of the change (increase/decrease). Such revisions would be a better companion for the figures.

We agree and have added these changes directly to the results section.

Given that ciliary MCHR1 expression is resilient to physiological or perhaps even pharmacological manipulation, it would be appropriate to speculate on the role of ciliary MCHR1 expression in the Discussion. Is ciliary MCH receptor expression required for signaling? Is the receptor expressed elsewhere on the cell?

We have added several ideas to the revised discussion regarding this point. For example, the following has been added: It is possible that in some cases, the pool of receptor that is signaling is on the plasma membrane and not the cilia membrane and thus, cilia localization appears stable for a given GPCR. Minor: Figure 2A is labeled MCHR1 when it appears to be the ligand, MCH

We have corrected this error.

Figure format - In the sample images of MCHR1, please add a pointer to indicate some examples of cilia in the image. Also, please outline the area that is shown in the higher magnification image. 4

We have added arrows and the outline of the inset to all data.

At the end of the third paragraph of the Results section the authors mention cilia frequency being unchanged under fasting or obesity and refer to data not shown. All data referenced in a manuscript should be shown for transparency and rigor.

We have added frequency data to all revised figures and have made all data available.

## References

[B1] Al-Massadi O, Dieguez C, Schneeberger M, López M, Schwaninger M, Prevot V, Nogueiras R (2021) Multifaceted actions of melanin-concentrating hormone on mammalian energy homeostasis. Nat Rev Endocrinol 17:745–755. 10.1038/s41574-021-00559-1 34608277

[B2] Alhassen W, Kobayashi Y, Su J, Robbins B, Nguyen H, Myint T, Yu M, Nauli SM, Saito Y, Alachkar A (2022) Regulation of brain primary cilia length by MCH signaling: evidence from pharmacological, genetic, optogenetic, and chemogenic manipulations. Mol Neurobiol 59:245–265. 10.1007/s12035-021-02511-w 34665407 PMC9083846

[B3] Balber T, Bencurova K, Kiefer FW, Kulterer OC, Klebermass EM, Egger G, Tran L, Wagner KH, Viernstein H, Pallitsch K, Spreitzer H, Hacker M, Wadsak W, Mitterhauser M, Philippe C (2019) In vitro radiopharmaceutical evidence for MCHR1 binding sites in murine brown adipocytes. Front Endocrinol (Lausanne) 10:324. 31244769 10.3389/fendo.2019.00324 PMC6581027

[B4] Bangs F, Anderson KV (2017) Primary cilia and mammalian hedgehog signaling. Cold Spring Harb Perspect Biol 9:a028175. 10.1101/cshperspect.a028175 27881449 PMC5411695

[B5] Bansal R, Engle SE, Kamba TK, Brewer KM, Lewis WR, Berbari NF (2021) Artificial intelligence approaches to assessing primary cilia. J Vis Exp (171):e62521. 10.3791/62521 PMC8791558 33999029

[B6] Becker-Krail DD, Walker WH 2nd, Nelson RJ (2022) The ventral tegmental area and nucleus accumbens as circadian oscillators: implications for drug abuse and substance use disorders. Front Physiol 13:886704. 35574492 10.3389/fphys.2022.886704 PMC9094703

[B7] Berbari NF, Lewis JS, Bishop GA, Askwith CC, Mykytyn K (2008a) Bardet-Biedl syndrome proteins are required for the localization of G protein-coupled receptors to primary cilia. Proc Natl Acad Sci U S A 105:4242–4246. 10.1073/pnas.0711027105 18334641 PMC2393805

[B8] Berbari NF, Johnson AD, Lewis JS, Askwith CC, Mykytyn K (2008b) Identification of ciliary localization sequences within the third intracellular loop of G protein-coupled receptors. Mol Biol Cell 19:1540–1547. 10.1091/mbc.e07-09-0942 18256283 PMC2291422

[B9] Berbari NF, O’Connor AK, Haycraft CJ, Yoder BK (2009) The primary cilium as a complex signaling center. Curr Biol 19:R526–R535. 10.1016/j.cub.2009.05.025 19602418 PMC2814769

[B10] Bishop GA, Berbari NF, Lewis J, Mykytyn K (2007) Type III adenylyl cyclase localizes to primary cilia throughout the adult mouse brain. J Comp Neurol 505:562–571. 10.1002/cne.21510 17924533

[B11] Blanco-Centurion C, Luo S, Spergel DJ, Vidal-Ortiz A, Oprisan SA, Van den Pol AN, Liu M, Shiromani PJ (2019) Dynamic network activation of hypothalamic MCH neurons in REM sleep and exploratory behavior. J Neurosci 39:4986–4998. 10.1523/JNEUROSCI.0305-19.2019 31036764 PMC6670248

[B12] Davenport JR, Watts AJ, Roper VC, Croyle MJ, van Groen T, Wyss JM, Nagy TR, Kesterson RA, Yoder BK (2007) Disruption of intraflagellar transport in adult mice leads to obesity and slow-onset cystic kidney disease. Curr Biol 17:1586–1594. 10.1016/j.cub.2007.08.034 17825558 PMC2084209

[B13] Dilsiz P, Aklan I, Sayar Atasoy N, Yavuz Y, Filiz G, Koksalar F, Ates T, Oncul M, Coban I, Ates Oz E, Cebecioglu U, Alp MI, Yilmaz B, Atasoy D (2020) MCH neuron activity is sufficient for reward and reinforces feeding. Neuroendocrinology 110:258–270. 10.1159/000501234 31154452

[B14] Diniz GB, Bittencourt JC (2019) The melanin-concentrating hormone (MCH) system: a tale of two peptides. Front Neurosci 13:1280. 31849590 10.3389/fnins.2019.01280 PMC6901935

[B15] Engle SE, Antonellis PJ, Whitehouse LS, Bansal R, Emond MR, Jontes JD, Kesterson RA, Mykytyn K, Berbari NF (2018) A CreER mouse to study melanin concentrating hormone signaling in the developing brain. Genesis 56:e23217. 10.1002/dvg.23217 29806135 PMC6167158

[B16] Engle SE, Bansal R, Antonellis PJ, Berbari NF (2021) Cilia signaling and obesity. Semin Cell Dev Biol 110:43–50. 10.1016/j.semcdb.2020.05.006 32466971 PMC8739279

[B17] Hastings MH, Maywood ES, Brancaccio M (2018) Generation of circadian rhythms in the suprachiasmatic nucleus. Nat Rev Neurosci 19:453–469. 10.1038/s41583-018-0026-z 29934559

[B18] Hsiao YC, Muñoz-Estrada J, Tuz K, Ferland RJ (2021) The transition zone protein AHI1 regulates neuronal ciliary trafficking of MCHR1 and its downstream signaling pathway. J Neurosci 41:3932–3943. 10.1523/JNEUROSCI.2993-20.2021 33741721 PMC8084322

[B19] Hwang SH, Mukhopadhyay S (2015) G-protein-coupled receptors and localized signaling in the primary cilium during ventral neural tube patterning. Birth Defects Res A Clin Mol Teratol 103:12–19. 10.1002/bdra.23267 24917297

[B20] Jasso KR, Kamba TK, Zimmerman AD, Bansal R, Engle SE, Everett T, Wu CH, Kulaga H, Reed RR, Berbari NF, McIntyre JC (2021) An N-terminal fusion allele to study melanin concentrating hormone receptor 1. Genesis 59:e23438. 10.1002/dvg.23438 34124835 PMC8376785

[B21] Kobayashi Y, Tomoshige S, Imakado K, Sekino Y, Koganezawa N, Shirao T, Diniz GB, Miyamoto T, Saito Y (2021) Ciliary GPCR-based transcriptome as a key regulator of cilia length control. FASEB Bioadv 3:744–767. 10.1096/fba.2021-00029 34485842 PMC8409570

[B22] Lee CH, Kang GM, Kim MS (2022) Mechanisms of weight control by primary cilia. Mol Cells 45:169–176. 10.14348/molcells.2022.2046 35387896 PMC9001153

[B23] Loktev AV, Jackson PK (2013) Neuropeptide Y family receptors traffic via the Bardet-Biedl syndrome pathway to signal in neuronal primary cilia. Cell Rep 5:1316–1329. 10.1016/j.celrep.2013.11.011 24316073

[B24] Messina MM, Boersma G, Overton JM, Eckel LA (2006) Estradiol decreases the orexigenic effect of melanin-concentrating hormone in ovariectomized rats. Physiol Behav 88:523–528. 10.1016/j.physbeh.2006.05.002 16793070

[B25] Mukhopadhyay S, Lu Y, Shaham S, Sengupta P (2008) Sensory signaling-dependent remodeling of olfactory cilia architecture in C. elegans. Dev Cell 14:762–774. 10.1016/j.devcel.2008.03.002 18477458 PMC2442577

[B26] Mukhopadhyay S, Wen X, Ratti N, Loktev A, Rangell L, Scales SJ, Jackson PK (2013) The ciliary G-protein-coupled receptor Gpr161 negatively regulates the Sonic hedgehog pathway via cAMP signaling. Cell 152:210–223. 10.1016/j.cell.2012.12.026 23332756

[B27] Nager AR, Goldstein JS, Herranz-Pérez V, Portran D, Ye F, Garcia-Verdugo JM, Nachury MV (2017) An actin network dispatches ciliary GPCRs into extracellular vesicles to modulate signaling. Cell 168:252–263.e14. 10.1016/j.cell.2016.11.036 28017328 PMC5235987

[B28] Olivier-Mason A, Wojtyniak M, Bowie RV, Nechipurenko IV, Blacque OE, Sengupta P (2013) Transmembrane protein OSTA-1 shapes sensory cilia morphology via regulation of intracellular membrane trafficking in C. elegans. Development 140:1560–1572. 10.1242/dev.086249 23482491 PMC3596995

[B29] Pal K, Hwang SH, Somatilaka B, Badgandi H, Jackson PK, DeFea K, Mukhopadhyay S (2016) Smoothened determines β-arrestin-mediated removal of the G protein-coupled receptor Gpr161 from the primary cilium. J Cell Biol 212:861–875. 10.1083/jcb.201506132 27002170 PMC4810300

[B30] Phua SC, Chiba S, Suzuki M, Su E, Roberson EC, Pusapati GV, Schurmans S, Setou M, Rohatgi R, Reiter JF, Ikegami K, Inoue T (2017) Dynamic remodeling of membrane composition drives cell cycle through primary cilia excision. Cell 168:264–279.e15. 10.1016/j.cell.2016.12.032 28086093 PMC5660509

[B31] Pissios P, Frank L, Kennedy AR, Porter DR, Marino FE, Liu FF, Pothos EN, Maratos-Flier E (2008) Dysregulation of the mesolimbic dopamine system and reward in MCH-/- mice. Biol Psychiatry 64:184–191. 10.1016/j.biopsych.2007.12.011 18281019

[B32] Presse F, Conductier G, Rovere C, Nahon JL (2014) The melanin-concentrating hormone receptors: neuronal and non-neuronal functions. Int J Obes Suppl 4:S31–S36. 10.1038/ijosup.2014.9 27152164 PMC4850588

[B33] Reiter JF, Leroux MR (2017) Genes and molecular pathways underpinning ciliopathies. Nat Rev Mol Cell Biol 18:533–547. 10.1038/nrm.2017.60 28698599 PMC5851292

[B34] Saito Y, Hamamoto A, Kobayashi Y (2013) Regulated control of melanin-concentrating hormone receptor 1 through posttranslational modifications. Front Endocrinol (Lausanne) 4:154. 24155742 10.3389/fendo.2013.00154 PMC3800845

[B35] Santollo J, Eckel LA (2008) The orexigenic effect of melanin-concentrating hormone (MCH) is influenced by sex and stage of the estrous cycle. Physiol Behav 93:842–850. 10.1016/j.physbeh.2007.11.050 18191424 PMC2573992

[B36] Schmidt P, Thomas L, Müller P, Scheidt HA, Huster D (2014) The G-protein-coupled neuropeptide Y receptor type 2 is highly dynamic in lipid membranes as revealed by solid-state NMR spectroscopy. Chemistry 20:4986–4992. 10.1002/chem.201304928 24623336

[B37] Shinde SR, Nager AR, Nachury MV (2020) Ubiquitin chains earmark GPCRs for BBSome-mediated removal from cilia. J Cell Biol 219:e202003020. 33185668 10.1083/jcb.202003020 PMC7716378

[B38] Simon A, Németh J, Jávor A, Komlósi I, Bai P, Oláh J, Juhász B, Kiss R, Szilvássy Z, Czeglédi L (2018) Feeding state and age dependent changes in melanin-concentrating hormone expression in the hypothalamus of broiler chickens. Acta Biochim Pol 65:251–258. 10.18388/abp.2017_2362 29850655

[B39] Singla V, Reiter JF (2006) The primary cilium as the cell's antenna: signaling at a sensory organelle. Science 313:629–633. 10.1126/science.1124534 16888132

[B40] Sun JS, Yang DJ, Kinyua AW, Yoon SG, Seong JK, Kim J, Moon SJ, Shin DM, Choi YH, Kim KW (2021) Ventromedial hypothalamic primary cilia control energy and skeletal homeostasis. J Clin Invest 131:e138107. 33021968 10.1172/JCI138107 PMC7773357

[B41] Tu H-Q, et al. (2022) Rhythmic cilium in SCN neuron is a gatekeeper for the intrinsic circadian clock. bioRxiv 477948. 10.1101/2022.01.26.477948.

[B42] Vaisse C, Reiter JF, Berbari NF (2017) Cilia and obesity. Cold Spring Harb Perspect Biol 9:a028217. 10.1101/cshperspect.a028217 28096262 PMC5495057

[B43] Wang J, Nikonorova IA, Gu A, Sternberg PW, Barr MM (2020) Release and targeting of polycystin-2-carrying ciliary extracellular vesicles. Curr Biol 30:R755–R756. 10.1016/j.cub.2020.05.079 32634412 PMC7668157

[B44] Wang J, Nikonorova IA, Silva M, Walsh JD, Tilton PE, Gu A, Akella JS, Barr MM (2021a) Sensory cilia act as a specialized venue for regulated extracellular vesicle biogenesis and signaling. Curr Biol 31:3943–3951.e3. 10.1016/j.cub.2021.06.040 34270950 PMC8440419

[B45] Wang L, Liu Y, Stratigopoulos G, Panigrahi S, Sui L, Zhang Y, Leduc CA, Glover HJ, De Rosa MC, Burnett LC, Williams DJ, Shang L, Goland R, Tsang SH, Wardlaw S, Egli D, Zheng D, Doege CA, Leibel RL (2021b) Bardet-Biedl syndrome proteins regulate intracellular signaling and neuronal function in patient-specific iPSC-derived neurons. J Clin Invest 131:e146287. 33630762 10.1172/JCI146287 PMC8262481

[B46] Wang Y, Bernard A, Comblain F, Yue X, Paillart C, Zhang S, Reiter JF, Vaisse C (2021c) Melanocortin 4 receptor signals at the neuronal primary cilium to control food intake and body weight. J Clin Invest 131:e142064. 33938449 10.1172/JCI142064 PMC8087202

[B47] Yamada Y, Post SR, Wang K, Tager HS, Bell GI, Seino S (1992a) Cloning and functional characterization of a family of human and mouse somatostatin receptors expressed in brain, gastrointestinal tract, and kidney. Proc Natl Acad Sci U S A 89:251–255. 10.1073/pnas.89.1.251 1346068 PMC48214

[B48] Yamada Y, Reisine T, Law SF, Ihara Y, Kubota A, Kagimoto S, Seino M, Seino Y, Bell GI, Seino S (1992b) Somatostatin receptors, an expanding gene family: cloning and functional characterization of human SSTR3, a protein coupled to adenylyl cyclase. Mol Endocrinol 6:2136–2142. 10.1210/mend.6.12.1337145 1337145

[B49] Yamada Y, Kagimoto S, Kubota A, Yasuda K, Masuda K, Someya Y, Ihara Y, Li Q, Imura H, Seino S (1993) Cloning, functional expression and pharmacological characterization of a fourth (hSSTR4) and a fifth (hSSTR5) human somatostatin receptor subtype. Biochem Biophys Res Commun 195:844–852. 10.1006/bbrc.1993.2122 8373420

[B50] Yasrebi A, Hsieh A, Mamounis KJ, Krumm EA, Yang JA, Magby J, Hu P, Roepke TA (2016) Differential gene regulation of GHSR signaling pathway in the arcuate nucleus and NPY neurons by fasting, diet-induced obesity, and 17β-estradiol. Mol Cell Endocrinol 422:42–56. 10.1016/j.mce.2015.11.007 26577678 PMC4742417

[B51] Ye F, Breslow DK, Koslover EF, Spakowitz AJ, Nelson WJ, Nachury MV (2013) Single molecule imaging reveals a major role for diffusion in the exploration of ciliary space by signaling receptors. Elife 2:e00654. 10.7554/eLife.00654 23930224 PMC3736543

[B52] Ye F, Nager AR, Nachury MV (2018) BBSome trains remove activated GPCRs from cilia by enabling passage through the transition zone. J Cell Biol 217:1847–1868. 10.1083/jcb.201709041 29483145 PMC5940304

[B53] Zamir N, Skofitsch G, Bannon MJ, Jacobowitz DM (1986) Melanin-concentrating hormone: unique peptide neuronal system in the rat brain and pituitary gland. Proc Natl Acad Sci U S A 83:1528–1531. 10.1073/pnas.83.5.1528 3513180 PMC323110

